# A B-cell developmental gene regulatory network is activated in infant AML

**DOI:** 10.1371/journal.pone.0259197

**Published:** 2021-11-18

**Authors:** Hamid Bolouri, Rhonda Ries, Laura Pardo, Tiffany Hylkema, Wanding Zhou, Jenny L. Smith, Amanda Leonti, Michael Loken, Jason E. Farrar, Timothy J. Triche, Soheil Meshinchi

**Affiliations:** 1 Center for Systems Immunology, Benaroya Research Institute, Seattle, WA, United States of America; 2 Clinical Research Division, Fred Hutchinson Cancer Research Center, Seattle, WA, United States of America; 3 Hematologics Inc., Seattle, WA, United States of America; 4 Van Andel Research Institute, Grand Rapids, MI, United States of America; 5 Arkansas Children’s Research Institute and University of Arkansas for Medical Sciences, Little Rock, AR, United States of America; Wayne State University, UNITED STATES

## Abstract

Infant Acute Myeloid Leukemia (AML) is a poorly-addressed, heterogeneous malignancy distinguished by surprisingly few mutations per patient but accompanied by myriad age-specific translocations. These characteristics make treatment of infant AML challenging. While infant AML is a relatively rare disease, it has enormous impact on families, and in terms of life-years-lost and life limiting morbidities. To better understand the mechanisms that drive infant AML, we performed integrative analyses of genome-wide mRNA, miRNA, and DNA-methylation data in diagnosis-stage patient samples. Here, we report the activation of an onco-fetal B-cell developmental gene regulatory network in infant AML. AML in infants is genomically distinct from AML in older children/adults in that it has more structural genomic aberrations and fewer mutations. Differential expression analysis of ~1500 pediatric AML samples revealed a large number of infant-specific genes, many of which are associated with B cell development and function. 18 of these genes form a well-studied B-cell gene regulatory network that includes the epigenetic regulators *BRD4* and *POU2AF1*, and their onco-fetal targets *LIN28B* and *IGF2BP3*. All four genes are hypo-methylated in infant AML. Moreover, micro-RNA *Let7a-2* is expressed in a mutually exclusive manner with its target and regulator *LIN28B*. These findings suggest infant AML may respond to bromodomain inhibitors and immune therapies targeting CD19, CD20, CD22, and CD79A.

## Introduction

**Fig 1:**
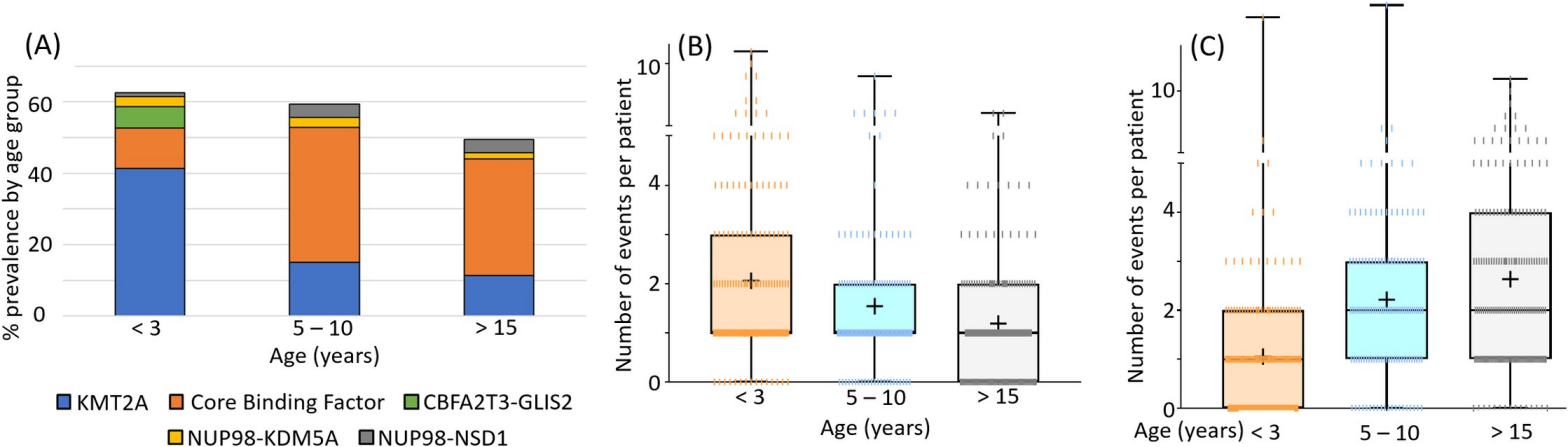
Infant AML is genomically distinct from other pediatric AML. Shown are three distinctive characteristics of infant AML in a total of 510 pediatric AML patients (<3 years, n = 184; 5 to 10 years, n = 140; >15 years, n = 186) from the CCG-2961, AAML03P1, and AAML0531 COG studies. (**A**) Infant AML is highly enriched in *KMT2A* (*MLL*) fusions, but has lower rates of Core Binding Factor (t(8;21) and inv(16) or t(16;16)) translocations. Certain fusions, such as *CBFA2T3*:*GLIS2* fusions appear exclusively in this age group. (**B**) Karyotypic events (translocations, and whole-chromosome and chromosome-arm level copy number alterations) are more prevalent in infant AML, with an average of 2.1, 1.5, and 1.2 events per patient for <3, 5 to 10, and >15 year old age groups, respectively; p = 0.0144 (<3 vs. 5 to 10) and p = <0.0001 (<3 vs. >15). (**C**) In contrast to structural/karyotypic alterations, infant AML is accompanied by few single nucleotide alterations and small insertions/deletions (indels), with averages of 1.1, 2.2, and 2.6 mutations per patient for the above described age groups, respectively; p = <0.0001 for each comparison of <3 versus other age groups.

In a recent large-scale study [1], we reported highly significant age-dependent effects in the genomic, epigenomic, and transcriptomic profiles of pediatric Acute Myeloid Leukemia (AML). AML in infants less than three years of age stands out in this respect. First, certain highly penetrant genomic translocations (e.g. *CBFA2T3*:*GLIS2*, **Fig 1A**) occur nearly exclusively in this group. Second, while the prevalence of structural alterations is higher in infants, there are remarkably few single nucleotide variants and indels per patient **(Fig 1B and 1C)**. Notwithstanding its low numbers of genomic alterations, infant AML comprises a diverse set of heterogeneous, poorly-understood disease-subtypes [2]. To better define the distinctive biology of infant AML, we carried out integrative analysis of genome-wide mRNA, miRNA, DNA methylation, and karyotype data in a cohort of approximately 1,500 pediatric AML samples.

## Methods

### Human subject details and sample preparation

All patient samples were obtained by member COG institutions after written consent from the parents/guardians of minors upon enrolling in the trial. The study was overseen by the Institutional Review Board at Fred Hutchinson Cancer Research Center (Protocol 9950). Selected clinical (e.g., age, presenting hematological indices, cytogenetic classification) and molecular features (e.g., *KIT*, *RAS*, *NPM1*, *WT1*, *CEBPA*, *IDH1* mutations, and *FLT3*/ITD allelic ratios) were clinically available for patients included in the NCI/TARGET cohort and are included in the clinical data file available at the TARGET data matrix.

Total RNA were extracted from ficoll-enriched, viably cryopreserved samples from the COG biorepository using the AllPrep Universal Extraction Kit (Qiagen). Nucleic acids were quantified by NanoDrop (Thermo Scientific). RNA samples were tested for quality and integrity using the Agilent 2100 Bioanalyzer (Agilent Technologies).

### RNA-seq for AAML1031

#### Data generation

Strand specific ribodepleted (RBD) libraries (version 2) from 1100 total RNA samples (1038 pediatric AMLs and 62 normal bone marrow controls) were constructed at the Genome Sciences Centre (GSC) at British Columbia Cancer Agency (BCCA, Vancouver, Canada). Libraries were pooled 4 per lane and each pool was sequenced on a single 75 base PET HiSeq 2500 lane. To remove cytoplasmic and mitochondrial ribosomal RNA (rRNA) species from total RNA NEBNext rRNA Depletion Kit for Human/Mouse/Rat was used (NEB, E6310X). Enzymatic reactions were set-up in a 96-well plate (Thermo Fisher Scientific) on a Microlab NIMBUS liquid handler (Hamilton Robotics, USA). 100ng of DNase I treated total RNA in 6 μL was hybridized to rRNA probes in a 7.5 μL reaction. Heat-sealed plates were incubated at 95oC for 2 minutes followed by incremental reduction in temperature by 0.1oC per second to 22oC (730 cycles). The rRNA in DNA hybrids were digested using RNase H in a 10 μL reaction incubated in a thermocycler at 37oC for 30 minutes. To remove excess rRNA probes (DNA) and residual genomic DNA contamination, DNase I was added in a total reaction volume of 25 μL and incubated at 37oC for 30 minutes. RNA was purified using RNA MagClean DX beads (Aline Biosciences, USA) with 15 minutes of binding time, 7 minutes clearing on a magnet followed by two 70% ethanol washes, 5 minutes to air dry the RNA pellet and elution in 36uL DEPC water. The plate containing RNA was stored at -80oC prior to cDNA synthesis.

First-strand cDNA was synthesized from the purified RNA (minus rRNA) using the Maxima H Minus First Strand cDNA Synthesis kit (Thermo-Fisher, USA) and random hexamer primers at a concentration of 5 μM along with a final concentration of 1 μg/uL Actinomycin D, followed by PCR Clean DX bead purification on a Microlab NIMBUS robot (Hamilton Robotics, USA). The second strand cDNA was synthesized following the NEBNext Ultra Directional Second Strand cDNA Synthesis protocol (NEB) that incorporates dUTP in the dNTP mix, allowing the second strand to be digested using USERTM enzyme (NEB) in the post-adapter ligation reaction and thus achieving strand specificity.

cDNA was fragmented by Covaris LE220 sonication for 55 seconds at a “Duty cycle” of 20% and “Intensity” of 5 to achieve 200–250 bp average fragment lengths. The paired-end sequencing library was prepared following the BC Cancer Agency Genome Sciences Centre strand-specific, plate-based library construction protocol on a Microlab NIMBUS robot (Hamilton Robotics, USA). Briefly, the sheared cDNA was subject to end-repair and phosphorylation in a single reaction using an enzyme premix (NEB) containing T4 DNA polymerase, Klenow DNA Polymerase and T4 polynucleotide kinase, incubated at 20oC for 30 minutes. Repaired cDNA was purified in 96-well format using PCR Clean DX beads (Aline Biosciences, USA), and 3’ A-tailed (adenylation) using Klenow fragment (3’ to 5’ exo minus) and incubation at 37oC for 30 minutes prior to enzyme heat inactivation. Illumina PE adapters were ligated at 20oC for 15 minutes. The adapter-ligated products were purified using PCR Clean DX beads, then digested with USERTM enzyme (1 U/μL, NEB) at 37oC for 15 minutes followed immediately by 13 cycles of indexed PCR using Phusion DNA Polymerase (Thermo Fisher Scientific Inc. USA) and Illumina’s PE primer set. PCR parameters: 98?C for 1 minute followed by 13 cycles of 98C 15 seconds, 65C 30 seconds and 72?C 30 seconds, and then 72?C 5 minutes. The PCR products were purified and size-selected using a 1:1 PCR Clean DX beads-to-sample ratio (twice), and the eluted DNA quality was assessed with Caliper LabChip GX for DNA samples using the High Sensitivity Assay (PerkinElmer, Inc. USA) and quantified using a Quant-iT dsDNA High Sensitivity Assay Kit on a Qubit fluorometer (Invitrogen) prior to library pooling and size-corrected final molar concentration calculation for Illumina HiSeq2500 sequencing with paired-end 75 base reads.

#### Data processing

Libraries were aligned to Hg19 (GRCh37-lite) reference genome using BWA v0.5.7 with default parameters, except with the addition of the "-s" option. Reads were discarded if they failed the Illumina chastity filter or a had phred mapping quality less than 10. Gene level coverage analysis was performed using the BCCA’s GSC pipeline v1.1, and composite gene models were generated using Ensembl v69 annotations.

### RNA-seq for the NCI/COG TARGET project AML samples

#### Data generation

Strand specific mRNA libraries from 466 total RNA samples (446 pediatric AML samples and 20 normal bone marrow controls) were constructed at BCCA’s GSC. Approximately 15 libraries were pooled and sequenced on 75 base PET lanes. Additional information on library preparation and specifications for sequencing can be found as previously described.^1^

#### Data processing

Briefly, Illumina paired-end RNA sequencing reads were aligned to GRCh37-lite genome-plus-junctions reference using BWA version 0.5.7. BWA was run using default parameters, except that the option (-s) was included to disable Smith-Waterman alignment. Reads failing the Illumina chastity filter were flagged with a custom script, and duplicated reads were flagged with Picard Tools. Gene, isoform, and exon-level quantification were performed as previously described. Transcriptomic data were de novo assembled using ABySS (v1.3.2) and trans-ABySS (v1.4.6). RNA-seq assembly alternate k-mers from k50-k96 were performed using positive strand and ambiguous stand reads as well as negative strand and ambiguous strand reads. The positive and negative strand assemblies were extended where possible, merged and then concatenated together to produce a meta-assembly contig dataset. Large scale rearrangements and gene fusions from RNA-seq libraries were identified from contigs that had high confidence GMAP (v2012-12-20) alignments to two distinct genomic regions. Evidence for the alignments were provided from aligning reads back to the contigs and from aligning reads to genomic coordinates. Additional parameters and specification can be found as previously described.

### miRNA-seq

#### Data generation

miRNA libraries from 1100 total RNA samples (1038 pediatric AML samples and 62 normal bone marrow controls) were constructed at BCCA’s GSC. Four libraries were pooled per lane and sequenced on a single short SET HiSeq 2500.

Small RNAs, containing microRNA (miRNA), in the flow-through material following mRNA purification on a MultiMACS separator (Miltenyi Biotec, Germany) are recovered by ethanol precipitation. MiRNA-seq libraries are constructed using a 96-well plate-based protocol developed at the BC Cancer Agency, Genome Sciences Centre. Briefly, an adenylated single-stranded DNA 3’ adapter is selectively ligated to miRNAs using a truncated T4 RNA ligase2 (NEB Canada, cat. M0242L). An RNA 5’ adapter is then added, using a T4 RNA ligase (Ambion USA, cat. AM2141) and ATP. Next, first strand cDNA is synthesized using Superscript II Reverse Transcriptase (Invitrogen, cat.18064 014), and serves as the template for PCR. Index sequences (6 nucleotides) are introduced at this PCR step to enable multiplexed pooling of miRNA libraries. PCR products are pooled, then size-selected on an in-house developed 96-channel robot to enrich the miRNA containing fraction and remove adapter contaminants. Each size-selected indexed pool is ethanol precipitated and quality checked on an Agilent Bioanalyzer DNA 1000 chip and quantified using a Qubit fluorometer (Invitrogen, cat. Q32854). Each pool is then diluted to a target concentration for cluster generation and loaded into a single lane of a HiSeq 2000 flow cell for sequencing with a 31-bp main read (for the insert) and a 7-bp read for the index.

#### Data processing

Data processing was carried out using the miRNA 3.0 protocol by BCCA’s GSC. In brief, reads were 31-bp in length and trimmed to remove adapter sequences; any trimmed reads shorter than 15bp were discarded. The trimmed reads for each sample were aligned to the NCBI GRCh37-lite using BWA v0.5.7 and duplicated reads were marked with Picard Tools. Only perfect alignments with no mismatches were retained for quantification. Reads were annotated using miRBase v20 and normalized to million miRNA-aligned reads (RPM).

### Flow cytometry

Bone marrow aspirates were collected in heparin (the preferred anti-coagulant) or EDTA. Briefly, 100 μL of bone marrow was added to cocktails of pre-titered antibodies at room temperature in the dark. Red blood cells were lysed using 3.5 mL of buffered NH4Cl (0.83%) at 37°C for 5 minutes before centrifugation at 300G. Cells were then washed with 3 mL of phosphate buffered saline containing 2% fetal calf serum and re-suspended to 0.5 mL in 1% paraformaldehyde for analysis on one of three FACS Calibur instruments (Becton Dickinson Biosciences, San Jose, CA).

200,000 events were collected for each tube (see Table 1). The flow cytometers were cross standardized and calibrated using RCP-30A and RFP-30A beads (Spherotech, Lake Forest, IL) with spectral compensation performed using peripheral blood cells labeled with CD4 (SK3, BD) conjugated to fluorescein (FITC), phycoerythrin (PE), peridinin chloro- phyll protein (PerCP) or allophycocyanin (APC). Eight combinations of antibodies used are presented in the table opposite.

**Table 1:** Flow cytometry markers per tube.

Tube 1	HLADR	CD11b	CD45	CD34
Tube 2	CD36	CD38	CD45	CD34
Tube 3	CD15	CD13	CD45	CD34
Tube 4	CD14	CD33	CD45	CD34
Tube 5	CD7	CD56	CD45	CD34
Tube 6	CD38	CD117	CD45	CD34
Tube 7	CD36	CD64	CD45	CD34
Tube 8	CD19	CD123	CD45	CD34

### RT qPCR

RNAs were normalized by concentration and reverse transcribed using Maxima™ H Minus cDNA Synthesis Master Mix (Thermo Scientific) per kit protocol. Each 20 μL qPCR reaction contained 50 ng cDNA equivalent, 10 μL TaqMan™ Universal PCR Master Mix (Applied Biosystems), and 1 μL 20X assay mix and were run in duplicate. Thermocycler conditions were set to 50°C for 2 minutes, 95°C for 10 minutes, followed by 40 cycles of 95°C for 15 seconds, and 60°C for 1 minute. Purchased assay mixes were BRD4 (TaqMan assay ID Hs04188087_m1), IGF2BP3 (TaqMan assay ID Hs00559907_g1), LIN28B (TaqMan assay ID Hs01013729_m1), POU2AF1 (TaqMan assay ID Hs01573371_m1), and GUSB endogenous control (TaqMan assay ID Hs99999908_m1) (Applied Biosystems). Plasmids used to generate copy number standards were purchased from DNASU Plasmid Repository and included LIN28B (Clone ID HsCD00515034), BRD4 (Clone ID HsCD00814539), and GUSB (Clone ID HsCD00044993). qPCR was carried out and results analyzed by comparative Ct or absolute quantification using a standard curve as appropriate using QuantStudio™ 5 Real-Time PCR System (Applied Biosystems).

### DNA-methylation

Bisulfite conversion of genomic DNA was performed with EZ DNA methylation Kit (Zymo Research, Irvine, CA) following the manufacturer’s protocol with modifications for the Infinium Methylation Assay. Briefly, one microgram of genomic DNA was mixed with 5 μl of Dilution Buffer and incubated at 37°C for 15 minutes and then mixed with 100 μl of conversion reagent prepared as instructed in the protocol. Mixtures were incubated in a thermocycler for 16 cycles at 95°C for 30 seconds and 50°C for 60 minutes. Bisulfite-converted DNA samples were loaded onto the provided 96-column plates for desulphonation, washing and elution. The concentration of bisulfite-converted, eluted DNA was measured by UV-absorbance using a NanoDrop-1000 (Thermo Fisher Scientific, Waltham, MA). Bisulfite-converted genomic DNA was analyzed using the Infinium MethylationEPIC Beadchip Kit (Illumina, San Diego, CA, #WG-317-1003). DNA amplification, fragmentation, array hybridization, extension and staining were performed with reagents provided in the kit according to the manufacturer’s protocol (Illumina Infinium II Methylation Assay, #WG-901-2701).

Briefly, 4 μl of bisulfite-converted genomic DNA at a minimum concentration of 20 ng/μL) was added to 0.8 ml 96-well storage plate (Thermo Fisher Scientific), denatured in 0.014N sodium hydroxide, neutralized and then amplified for 20–24 hours at 37oC. Samples were fragmented at 37°C for 60 minutes and precipitated in isopropanol. Re-suspended samples were denatured in a 96-well plate heat block at 95°C for 20 minutes. 15 μl of each sample was loaded onto an 8-sample BeadChip, assembled in the hybridization chamber as instructed by the manufacturer and incubated at 48°C for 16–20 hours. Following hybridization, the BeadChips were washed and assembled in a fluid flow-through station for primer-extension reaction and staining with reagents and buffers provided. Polymer-coated BeadChips were scanned in an iScan scanner (Illumina) using Inf Methylation mode.

### Karyotyping

Karyotypic information using the International System for Human Cytogenetic Nomenclature (ISCN) is gathered on all AML patients enrolled in COG clinical trials and centrally reviewed. In addition, Florescence In Situ Hybridization (FISH) probes are commonly employed to confirm cytogenetic alterations. All computationally predicted fusions derived from the transcriptome data were compared against karyotypic information.

### Statistical tests

Differential mRNA expression was estimated using the Bioconductor/R ‘limma’ package. For miRNAs, differential expression was determined using the Wilcoxon/Mann-Whitney test. Differential mRNA and miRNA expression p-values reported are adjusted for multiple-hypothesis testing using the Bonferroni-Holm method. Additional p-values are reported for t-tests and Pearson correlations as specified in the text.

## Results

### Overview

Using newly generated mRNA and miRNA expression data at diagnosis for 1,038 pediatric AML samples from the Children’s Oncology Group (COG) AAML1031 study (ClinicalTrials.gov Identifier: NCT01371981), we compared infant AML to AML in children older than 5 years. We discovered that many of the most significantly up-regulated genes in infant AML are associated with B cell development and function. A similar pattern was observed in RNA-seq data for 446 additional samples from the NCI/COG TARGET project.

Of special note, mRNA expression of a subset of 56 genes sharply segregates infant AML from older age groups. Fifty-one of these genes are B-cell associated, and 18 of these genes form a well-studied gene regulatory network (**GRN**) normally associated with B cell development. In spite of the genetic heterogeneity among infant AML, this B cell GRN is up-regulated in most infant AML cases, suggesting that its activity is a common feature of infant AML.

The genes in this GRN include the epigenetic ‘reader’ *BRD4* and its known targets, *LIN28B*, *IGF2BP3*, and *POU2AF1*. In addition, *POU2AF1* recruits the histone lysine demethylase *JMJD1* to epigenetically activate downstream target genes [3]. Differential DNA methylation analysis in 423 samples (98 infants, 325 children older than 5) revealed that several genes in this B cell GRN, including *BRD4* and *POU2AF1*, are hypo-methylated in infants.

### Multiple B-cell associated genes are up-regulated in infant AML

Comparison of the expression profiles of infant AML (n = 250) to AML in patients greater than five years of age (n = 706) in the COG AAML1031 cohort revealed 6,176 differentially expressed mRNAs and 703 differentially expressed miRNAs between the two age groups (**S1 and S2 Tables**). Although cytogenetic aberrations impact the expression of specific genes (**S1 Fig**), the gene expression differences reported here are age-specific and span cytogenetic sub-types (discussed below).

**Fig 2:**
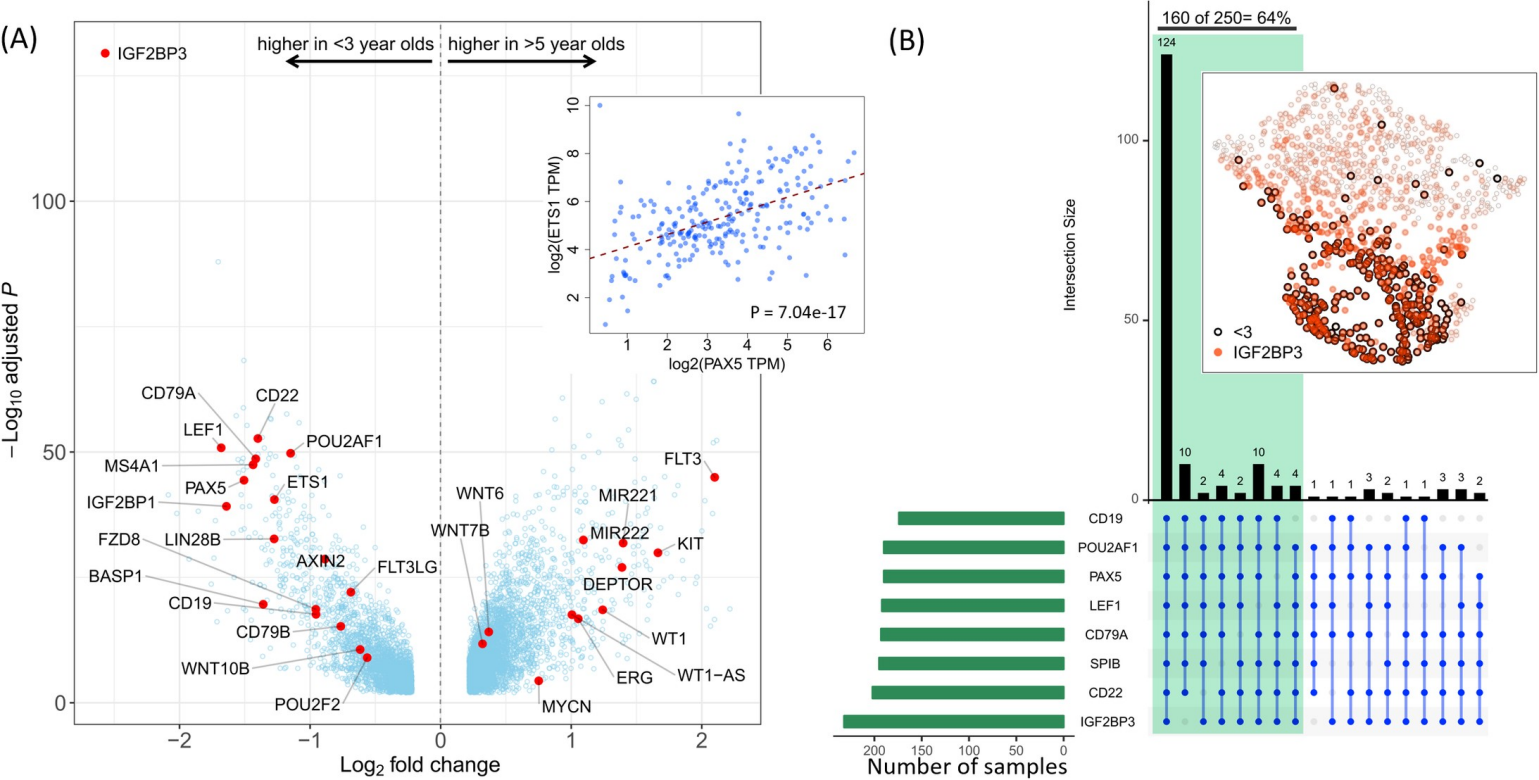
Many B cell specific genes are differentially expressed genes in infant AML. (**A**) Volcano plot showing–log10(adjusted p-value) and log2(fold change) of genes differentially expressed between infant AML (n = 250) and AML in children >5 years old (n = 706). Genes to the left of the plot are up-regulated in infant AML compared to AML >5 year olds. Genes to the right of the plot are down-regulated in infant AML. Some notable genes are labeled individually. Canonical WNT signaling genes *WNT10B*, *FZD6*, *LEF1*, and *AXIN2* are up-regulated in infant AML, as are the transcription factor *ETS1* and a large number of B-cell genes. In contrast, the AML associated genes *KIT*, *FLT3*, and *WT1* are down-regulated in infants compared to children >5 years old. The inset shows a high correlation between the B-cell marker *PAX5*, and its transcriptional co-activator *ETS1*. (**B**) A group of eight B-cell associated infant AML genes (listed at bottom-left) are expressed at above average (total cohort size = 1,038) levels in ~ 64% of infant AML (marked by the green shaded area). For each gene, green bars at left show the numbers of infant AML cases with above average expression. Connected blue disks below the top bar plot indicate combinations of genes and the corresponding black bars indicate the number of samples over-expressing that combination of genes. Inset shows a 2-dimensional UMAP projection of the expression data, in which each point represents the expression profile of one sample for the eight genes. Infants are marked by a black circle, and intensity of red indicates the expression level of IGF2BP3 per sample.

Of note, components of canonical WNT signaling (*FZD8*, *LEF1*, *WNT10B*, *AXIN2*), the ETS1 transcription factor and its co-activator PAX5 [4], and the stem-cell and fetal B cell developmental gene *LIN28B* [5, 6] are expressed at distinctly higher levels in infants compared to older children with AML (**Fig 2A**, inset shows high correlation between *PAX5* and *ETS1* mRNA levels).

We do not find a significant difference in the prevalence of ETS-family fusions across age groups in pediatric AML. Moreover, the *WT1* gene, which is frequently up-regulated in pediatric AML [7] and can activate *ETS1* [8], is down-regulated in infant AML (Fig 2A). Consistent with this finding, *BASP1*, which has been shown to block *WT1* mediated activation of *ETS1* [8], is highly up-regulated in infant AML (Fig 2A).

Remarkably, at least six of eight B cell associated genes are expressed at above average levels in 160 of 250 (64%) infants in our cohort (**Fig 2B**). Accordingly, unsupervised dimension reduction using Uniform Manifold Approximation and Projection (UMAP) [9] revealed that these eight genes segregate infant AML patients into a distinct cluster (**inset, Fig 2B**).

**Fig 3:**
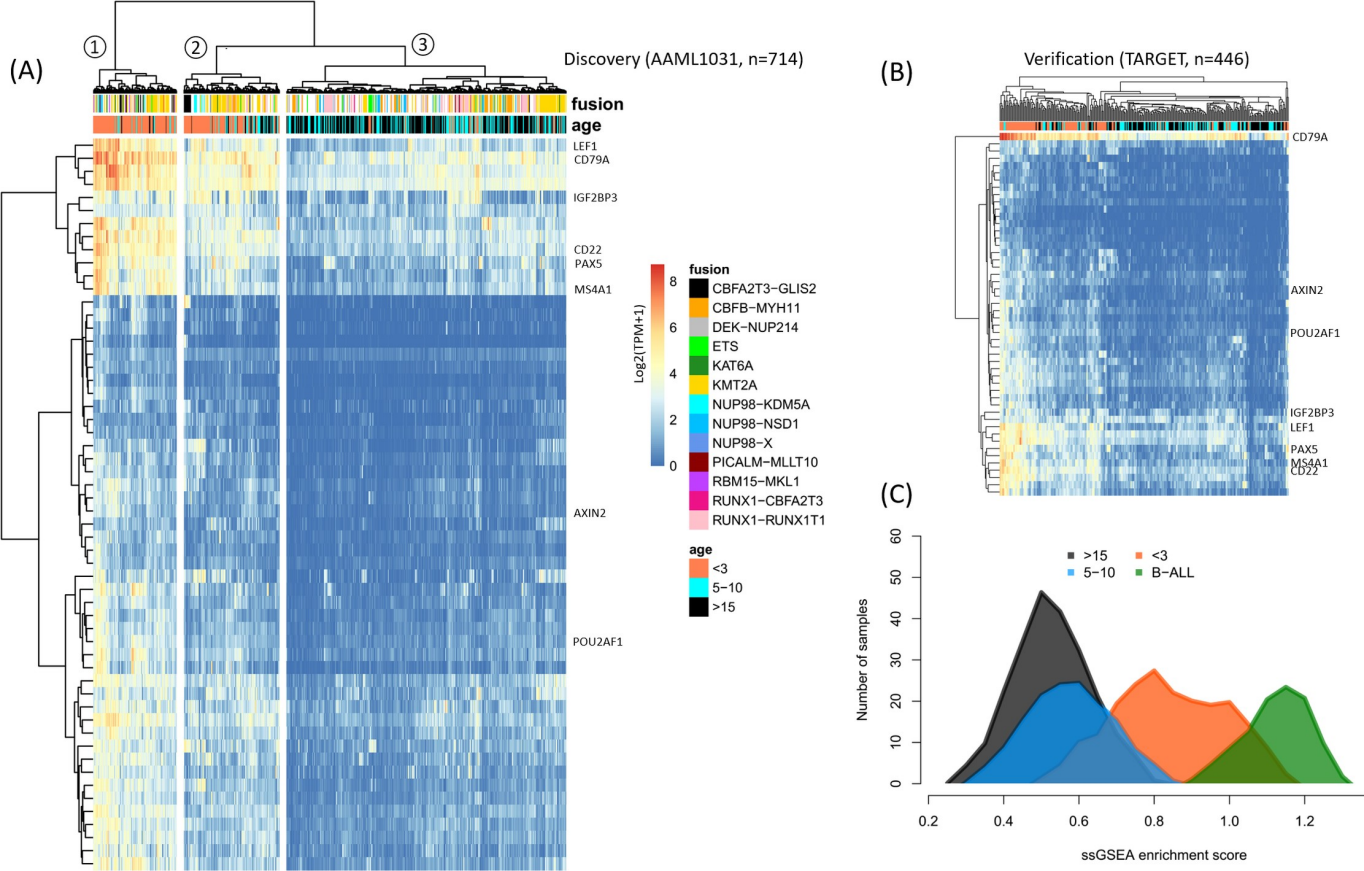
A group of 56 genes (51 of which are B-cell associated) clusters pediatric AML by age. (**A**) Heatmap showing unsupervised hierarchical clustering using Euclidian distance of log2(TPM+1) values and “ward.D2” clustering (n = 714 patients falling within the age-groups shown). Genes of special note are underlined in red. Age groups and translocations (fusions) are indicated in the annotation rows at the top. (**B**) Unsupervised hierarchical clustering in an independent cohort of 446 pediatric AML patients produces a similar pattern of clustering by age groups. (**C**) Single Sample Gene Set Enrichment Analysis (ssGSEA) enrichment scores (horizontal axis) using the 56 genes in (A) suggests the expression pattern of these genes in infant AML is highly-similar–but not identical–to B-ALL from the NCI TARGET project.

Further analysis revealed a group of 56 genes that are highly correlated with age (Pearson correlation coefficient > 0.375). Remarkably, 51 of these genes (91%) are B cell associated, and 32 (57%) are putative ETS1 targets (**S3 and S4 Tables)**. Unsupervised hierarchical clustering using these 56 genes generated three distinct clusters of patients (**Fig 3A**): Cluster 1 is almost entirely made up of infants, Cluster 2 is highly enriched in infants, while Cluster 3 is nearly devoid of infants. Unsupervised hierarchical clustering of 446 additional samples from the NCI/COG TARGET project [1] similarly grouped patients by age (**Fig 3B**).

Single sample Gene Set Enrichment Analysis (ssGSEA) scores for the above 56 genes revealed that although AML in children and young adults is highly distinct from B-ALL, infant AML is highly similar to infant B-ALL (**Fig 3C**). We note however, that infant AML and B-ALL expression patterns are nonetheless distinct.

### Micro RNA expression in infant AML supports B-cell gene signature

**Fig 4:**
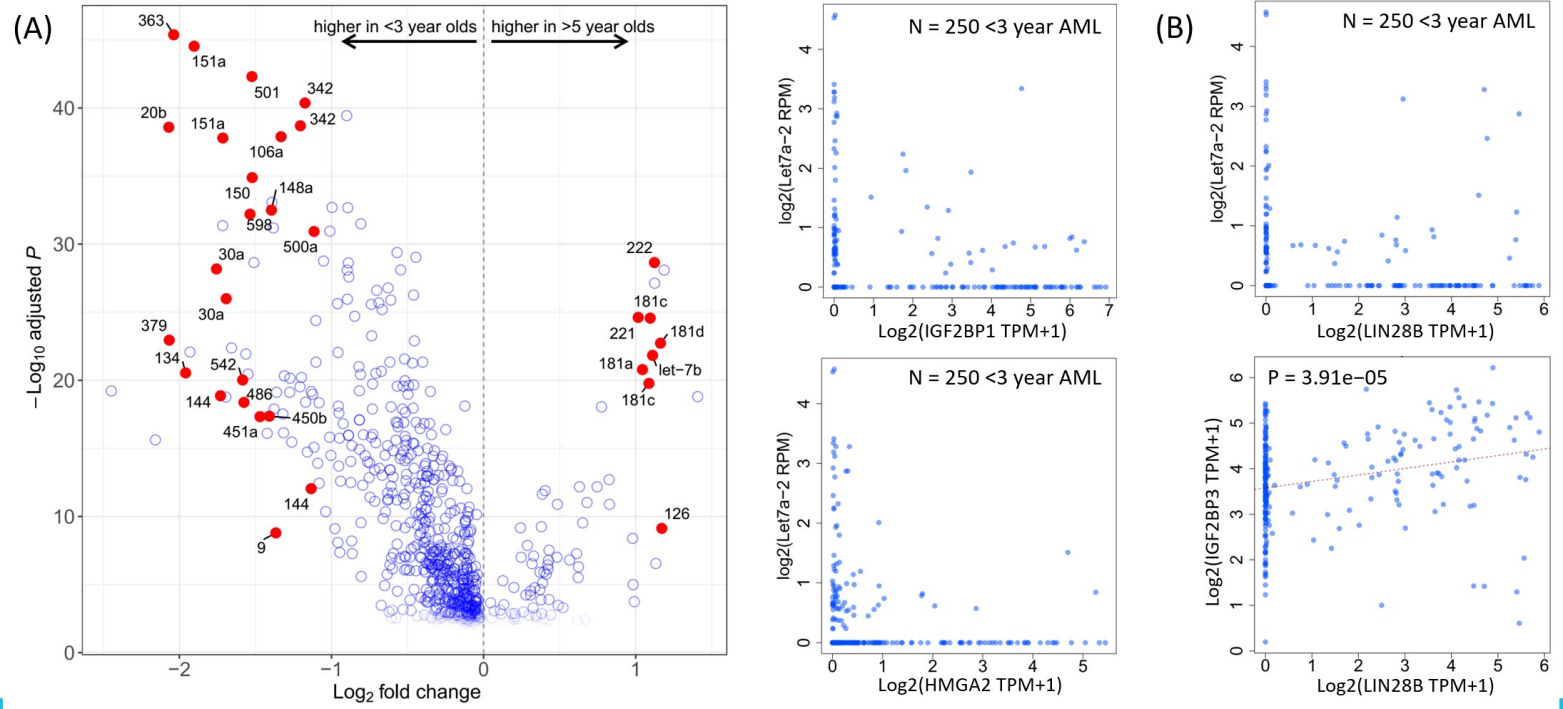
Micro RNAs in infant AML. (**A**) Volcano plot showing differentially expressed miRNAs in infants versus children >5 years old. miRNAs to the left of the plot are up-regulated in infant AML. Some notable miRNAs are labeled individually. (**B**) The *Let7* family of miRNAs is down-regulated in infant AML. Among *Let7* family members, *Let7a-2* exhibits particularly strong patterns of mutual exclusion with its putative target genes *IGF2BP1*, *LIN28B*, and *HMGA2*. Consistent with *Let7* repression of IGF2BPs, *LIN28B* expression is correlated with *IGF2BP3* expression.

Our ribosomal RNA-depleted RNA-seq data revealed a number of differentially regulated micro RNAs in infants compared to older AML patients. To clarify the role of miRNAs in infant AML, we analyzed genome-wide miRNA-seq expression data generated for the same cohort. As illustrated in **Fig 4A**, a distinctively large number of miRNAs are up-regulated in infant AML compared to AML in older children. *Let7-b* was previously reported to be repressed by *MLL* (*KMT2A*) gene rearrangements in infant ALL (PMID:22918121), and miR-9 has been shown to be up-regulated by *MLL* fusions in adult AML (PMID: 23798388). Among infant-specific miRs, *miR-150* has been shown to block B cell differentiation [10], while *miR-151* is over-expressed in germinal-center Diffuse Large B cell Lymphomas [11].

Interestingly, two of the most infant-specific miRNAs, *miR20b* and *miR363* are part of an X chromosome miR cluster that is upregulated in T cell leukemias [12], and represses the E2F1 transcription factor [13, 14], a cell-cycle regulator that is highly expressed in normal bone marrow and down-regulated in *KMT2E*-deleted AML [15].

Among the Let7 family of micro RNAs, *Let7a-2* shows a strong mutual exclusion with its putative targets *LIN28B* (which reciprocally represses Let7 miRs [16]), *IGF2BP1*, and *HMGA2* (**Fig 4B**). In addition, consistent with previous findings that *Let7* and *LIN28B* form a mutual repression loop upstream of IGFBPs, *IGF2BP3* expression is strongly correlated with *LIN28B* expression in a subset of infant AML (Fig 4B).

**Fig 5:**
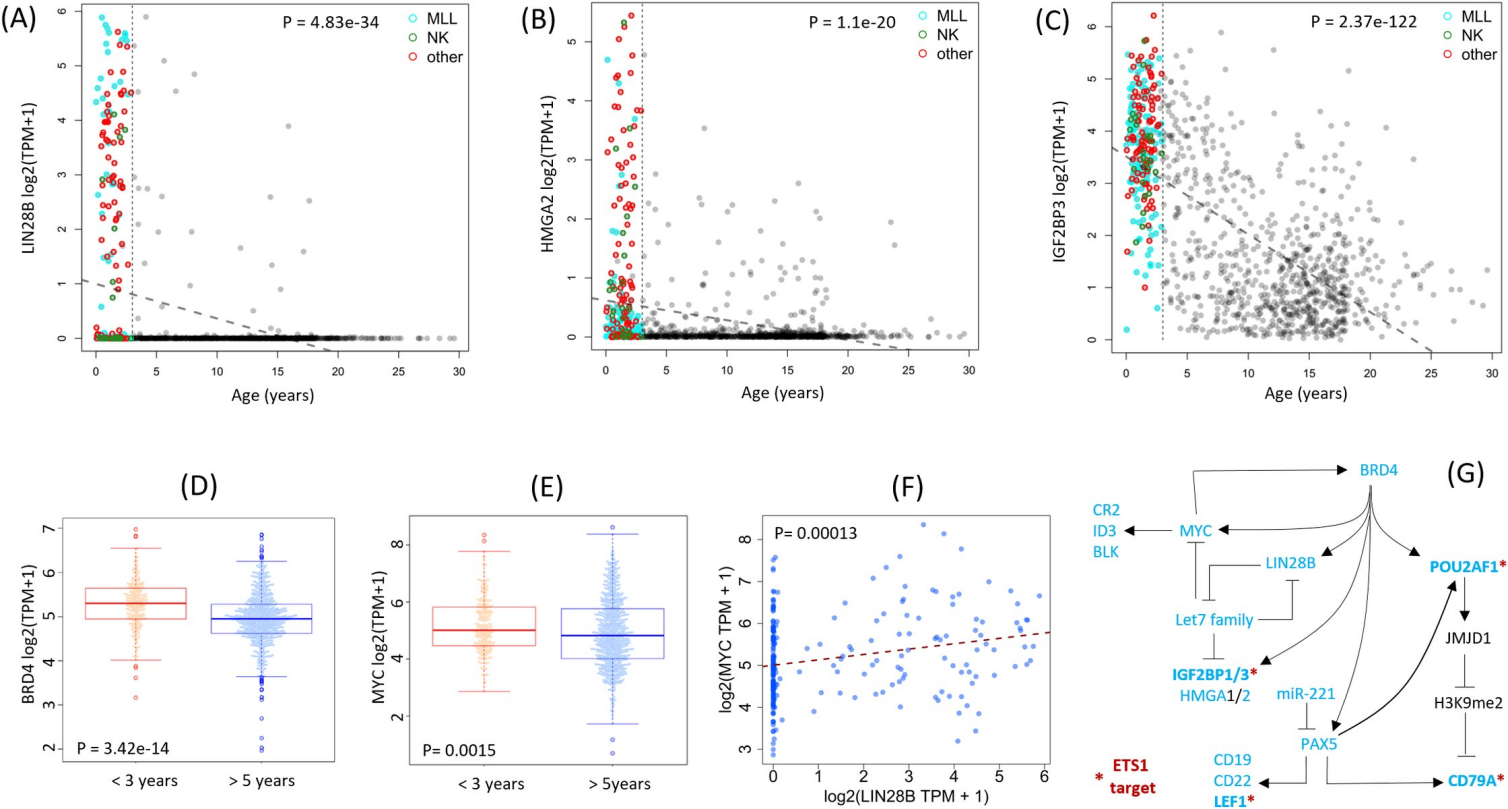
Up-regulation of a B-cell developmental gene regulatory network in infant AML. (**A-C**) Upregulation of *LIN28B* and its target genes *HMGA2* and *IGF2BP3* in infant AML. (**D, E**) *BRD4* and *MYC*, which can form an auto-regulatory loop, are both up-regulated in infant AML, and there is a concomitant positive correlation between *LIN28B* and *MYC* expression (**F**). (**G**) Genes in a well-studied set of gene regulatory interactions involved in B-cell development are differentially regulated (cyan colored nodes) in AML. Genes marked by * are also known targets of *ETS1*, which is also highly up-regulated in infant AML.

As shown in **Fig 5A–5C**, high expression levels of *LIN28B* and *HMGA2* are almost completely exclusive to <5 year olds, and nearly all infants express high levels of the onco-fetal gene *IGF2BP3* [17]. Remarkably, these high levels of expression occur regardless of the presence or type of chromosomal translocation.

Of particular interest, the expression of *BRD4*, a known epigenetic activator of *LIN28B* and IGF2BPs [16, 18], as well as the infant AML signature genes *PAX5* and *POU2AF1*, is significantly up-regulated in infants compared to >5 year olds with AML (**Fig 5D**). In a mouse *MLL-AF9* fusion model of AML, an RNAi screen previously identified BRD4 as a potential therapeutic target [19], and in the RN2 cell line derived from these mice, ETS-family DNA-binding motifs are enriched in BRD4 ChIP-seq peaks [20].

Bromodomain factors, LIN28B, Let7 miRs, and the MYC transcription factor form a self-stabilizing positive feedback loop that is activated in diverse cancers [5]. Consistent with a role for this regulatory network in infant AML, *MYC* expression is higher in infant AML (**Fig 5E**) and is correlated with the expression of *LIN28B* (**Fig 5F**).

**Fig 5G** summarizes the proposed gene regulatory network up-regulated in infant AML. Consistent with the up-regulation of *PAX5* in this network, the expression of the known *PAX5* repressor *miR-221* is down-regulated in infant AML (Fig 4A). In support of this finding, PAX5 was recently found to be a key regulator of CLL super enhancers essential for CLL cell survival [21]. Moreover, consistent with the large number of ETS targets in our infant-AML 56-gene signature, at least five genes in this network (*IGF2BP1/3*, *LEF1*, *POU2AF1*, *CD79A*) are also targeted by the infant-AML specific factor ETS1.

### Hypomethylation of key B-cell associated genes in infant AML

**Fig 6:**
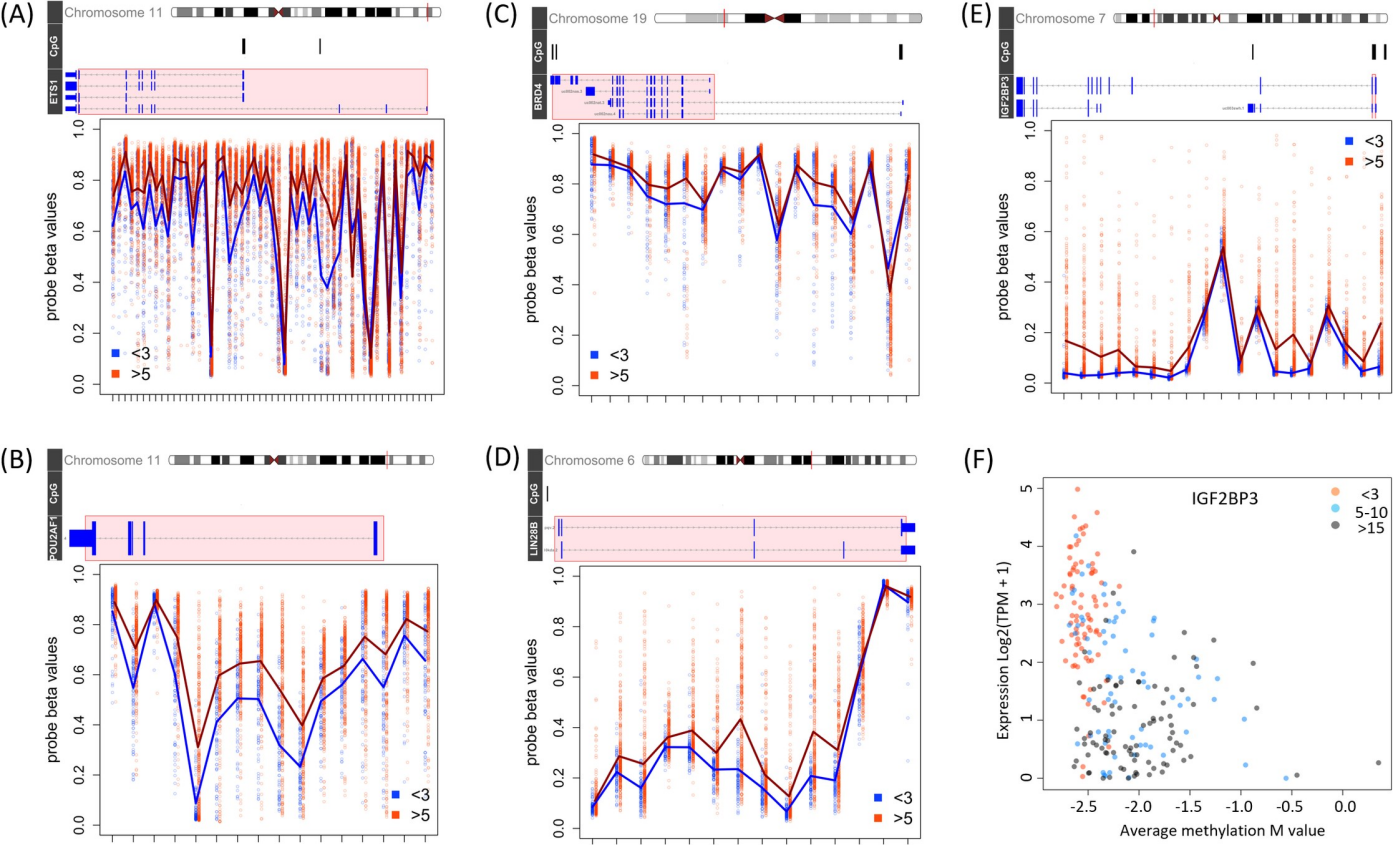
Five B-cell associated genes up-regulated in infant AML are hypomethylated at the DNA level. (**A-E**) Shown are differentially methylated region plots for *ETS1*, *BRD4*, *IGF2BP3*, *POU2AF1*, and *LIN28B*. The chromosome ideogram at the top shows the genomic location considered (red bar) in hg19 coordinates. The genome browser tracks below each ideogram show the locations of known CpG islands (black bars), and all UCSC transcript isoforms (horizontal lines, with intersecting blue bars marking exons). The super-imposed red box marks the region with differentially methylated probes displayed in detail in the panel below. The vertical axis in the differential methylation panels indicates fractional probe methylation level. CpG probes are organized in their chromosomal order along the horizontal axis. The red and blue plot points mark methylation levels in individual samples (blue = infants, red = >5 year olds). The red and blue lines connect the mean values of CpG methylation levels per patient group. (**F**) The expression if *IGF2BP3*, which is only differentially-methylated in a promoter CpG island region (panel E), is highly anti-correlated with the average methylation of all *IGF2BP3* CpG probes in this region and distinctive in infant AML (red plot points).

Given that BRD4 and POU2AF1 can activate their target genes epigenetically, we evaluated the DNA-methylation status of infant-specific AML genes in 423 cases (98 infants and 325 children older than 5 years). As shown in **Fig 6**, *BRD4* and its putative targets *LIN28B*, *IGF2BP3*, and *POU2AF1* all show highly significant patterns of de-methylation in infants (Bonferroni-Holm adjusted p-values for all shown CpG probes are < 0.05), as does *ETS1*, which is highly up-regulated in infant AML (Fig 2) and targets half of the genes in our infant AML signature. A full list of all genes with differentially methylated regions is given in **S4 Table**.

*POU2AF1* hypo-methylated probes span a known BRD4-regulated super-enhancer activated in Diffuse Large B Cell Lymphoma [22]. Hypo-methylated probes span the lengths of *ETS1*, *LIN28B*, and *BRD4* transcripts, consistent with having a functional role. For *IGF2BP3*, the 19 hypo-methylated probes fall in a single CpG island/shores region spanning exon and intron 1. Consistent with a functional role, the average methylation of the *IGF2BP3* CpG probes in this region is highly age-specific, with infant AML showing the highest expression and lowest methylation in a distinct cluster of patients (**Fig 6F**).

### Functional validation of B-cell signature genes in infant AML

**Fig 7:**
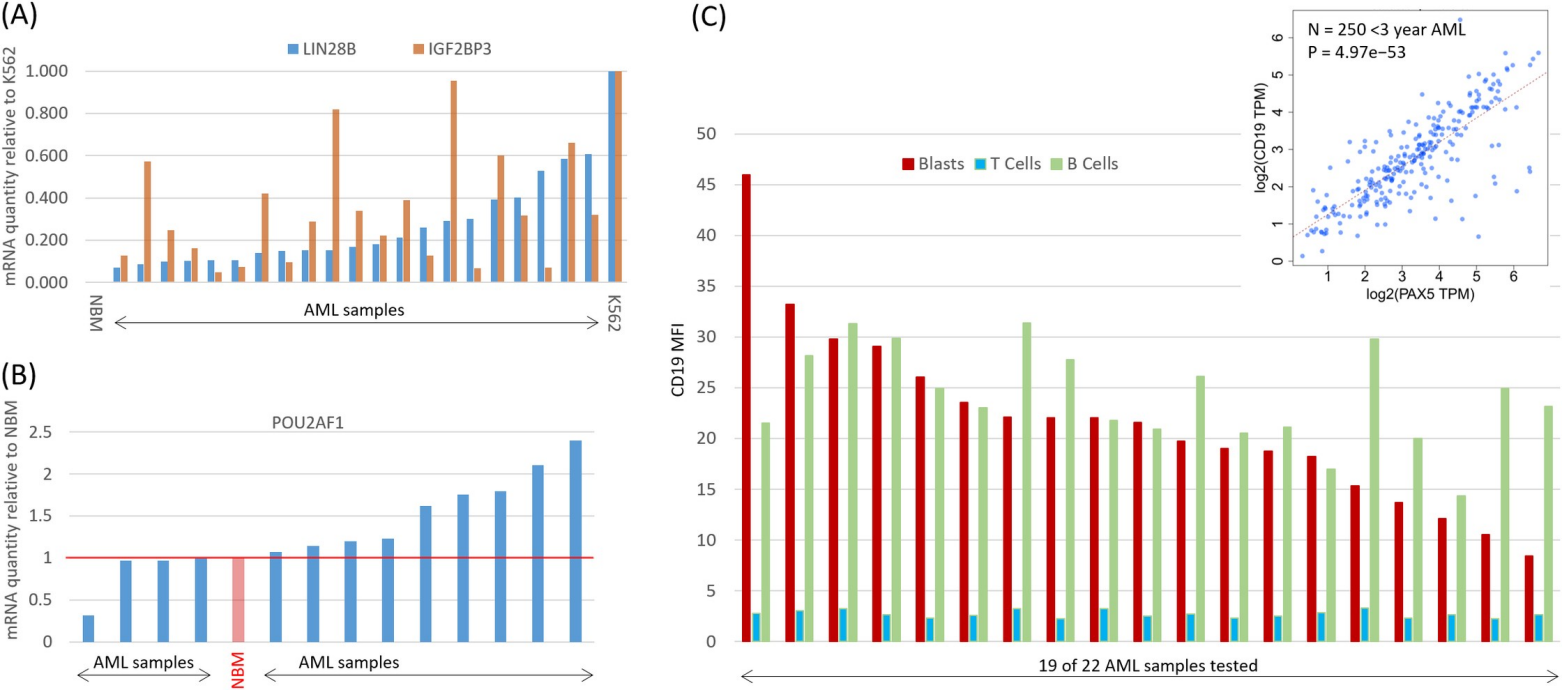
B-cell associated genes in infant AML are expressed at functionally significant levels. (**A, B**) *LIN28B*, *IGF2BP3*, and *POU2AF1*, are expressed at physiologically meaningful levels in infant AML. Shown are real-time quantitative PCR (RT qPCR) mRNA abundance measurements in 23 infant AML samples (22 for *POU2AF1*) compared to K562 cells. For comparison, expression in a pool of seven normal bone marrow samples is also shown. (**C**) The expression of CD19 protein in infant AML blasts is at levels comparable to that of B cells. Shown are flow-cytometry mean fluorescence intensity (MFI) values for AML blasts, T cells and B cells from 19 of 22 infant AML samples successfully tested. Inset shows the high correlation of the *PAX5* gene and its putative target *CD19*.

We used RT q-PCR to verify that key genes in our infant AML signature are expressed at physiologically significant levels. *POU2AF1* is expressed at significant levels in normal bone marrow (proteinatlas.org/ENSG00000110777-POU2AF1/tissue) and infant AML. As shown in **Fig 7**, *LIN28B*. *POU2AF1*, and *IGF2BP3* are all expressed at significant levels in the infant AML samples tested.

*LIN28B* is a key regulator of fetal hematopoiesis, but its expression is sharply down-regulated within days after birth [23]. Indeed, *LIN28B* expression in adult tissues is associated with 20 different human cancers [5]. High expression of *LIN28B* was previously reported in a fetal-like subset of juvenile myelomonocytic leukemia [24], and in KIT^+^ *MLL*-fusion mice and cell lines [16]. In support of a functional role for *LIN28B* in infant AML, we estimate that in infant AML *LIN28B* is expressed at approximately 10–60% of the level of its expression in K562 cells (**Fig 7**). K562 cells are reported to have > 160,000 molecules LIN28B mRNA per cell [25].

## Discussion

Emerging data indicate that AML in infancy has a distinct biology with a unique constellation of chromosomal translocations and paucity of other sequence variants, suggesting unique and presently untapped therapeutic vulnerabilities. Here, we verify the unique biology of AML in infants by demonstrating an infant AML expression signature that more closely resembles that of B ALL than AML in older children. Identification of a gene expression signature and associated gene regulatory network up-regulated in a large proportion of infant AML patients is unexpected and surprising given the genetic heterogeneity of this group, but may indicate convergence on a common and unique developmental pathway. Among the genes up-regulated in infant AML, *IGF2BP3*, and *LIN28B* are onco-fetal genes not normally expressed in blood/marrow. Other genes up-regulated in infant AML blasts are typically expressed only in B cells, although it is important to note that we also see up-regulation of other known IGF2BP3 target genes such as MMP9 [26], p ~ 1.64e-06.

We note that because *BRD4*, *LIN28B*, *Let7* miRs, and *MYC* form a self-reinforcing positive feedback loop, diverse perturbations with large effects (e.g. ETS-family fusions) or small effects (e.g. low copy-number alterations, regulatory DNA variations, or environmental factors) may be sufficient to inappropriately activate this GRN in an otherwise molecularly heterogeneous group of patients.

## Conclusions

Our findings have important clinical implications. Firstly, up-regulation of *BRD4* and its targets in infant AML suggest Bromodomain inhibitors may be effective in this patient group. Second, our 56-gene infant AML gene expression signature includes cell surface proteins such as *CD20* (*MS4A1*), *CD22*, and *CD79A*, suitable as immunotherapy targets. Antibodies targeting CD20 have proven effective in Chronic Lymphoblastic Leukemia [27]. CD79 antibodies were previously developed to address autoimmunity [28], and CAR-T cells targeting CD22 have shown promise in pre-B cell Acute Lymphoblastic Leukemia [29].

In addition, demonstration and validation of an ALL-like infant AML immunophenotype that includes elevated *CD19* expression provides an opportunity for CD19-targetting CAR-T cells, which have shown remarkable success in ALL and other blood disorders [30]. *PAX5* is a well-known upstream regulator of *CD19* that is up-regulated in infant AML (Figs 2 and 3). *CD19* and *PAX5* expression are highly correlated in infant AML (**inset of Fig 7C**). We confirmed that up-regulation of *CD19* occurs specifically in AML blasts by measuring blast-specific CD19 protein levels using multi-parameter flow cytometry. In 22 samples tested, 19 samples (86.4%) showed AML blast CD19 protein levels comparable to those of B cells (**Fig 7C,** see **S2 Fig for gating scheme**). Thus, a large proportion of infant AML patients may be suitable for CD19 CAR-T cell therapy.

## Supporting information

S1 Fig HOXA family gene expression in diagnostic samples with MLLT10 fusions. (TIF)

S2 Fig Flow Cytometry gates and scatter plots for one representative AML sample and an example normal bone marrow. (**A**) Left to right: CD45/Side Scatter plot shows cell composition of the specimen with abnormal blasts in red, normal neutrophils in blue and normal lymphocytes in grey. Blast cells (in red) were gated to show expression of the stem/progenitor marker CD34, and myeloid associated markers CD13, CD33 and CD117. The last panel shows CD19 and CD123 expression of the leukemic blast cells in red, relative to normal lymphocytes (B lymphocytes CD19 positive) and T lymphocytes (CD19 negative) in blue. (**B**) Left to right: CD45/Side Scatter plot showing composition of a normal BM specimen with CD34+ myeloid and lymphoid precursors in red, neutrophils in blue, monocytes in green and lymphoid precursors and mature lymphocytes in grey. CD34+ precursors (red) were gated and the expression of myeloid associated antigens CD13, CD33 and CD117 displayed vs CD34. Note there is no expression of either of these antigens on CD34+ lymphoblasts. The last plot shows CD19 and CD123 expression of CD34+ myeloblasts in red relative to normal T and B lymphocytes.
(TIF)

S1 Table Genes differentially expressed between <3 year infants with AML and >5 year children with AML. (XLSX)

S2 Table miRNAs differentially expressed between <3 year infants with AML and >5 year children with AML., and predicted targets of the top 31 miRs (sheet 2). (XLSX)

S3 Table Marker gens in <3 year infants with AML. (XLSX)

S4 Table Differentially methylated genes between <3 year infants with AML and >5 year children with AML. (XLSX)
