# Supplementary figures and images for: A B-cell developmental gene regulatory network is activated in infant AML
Source: PLoS One. 2021 Nov 18;16(11):e0259197. doi: 10.1371/journal.pone.0259197 (PMC8601427; doi:10.1371/journal.pone.0259197)

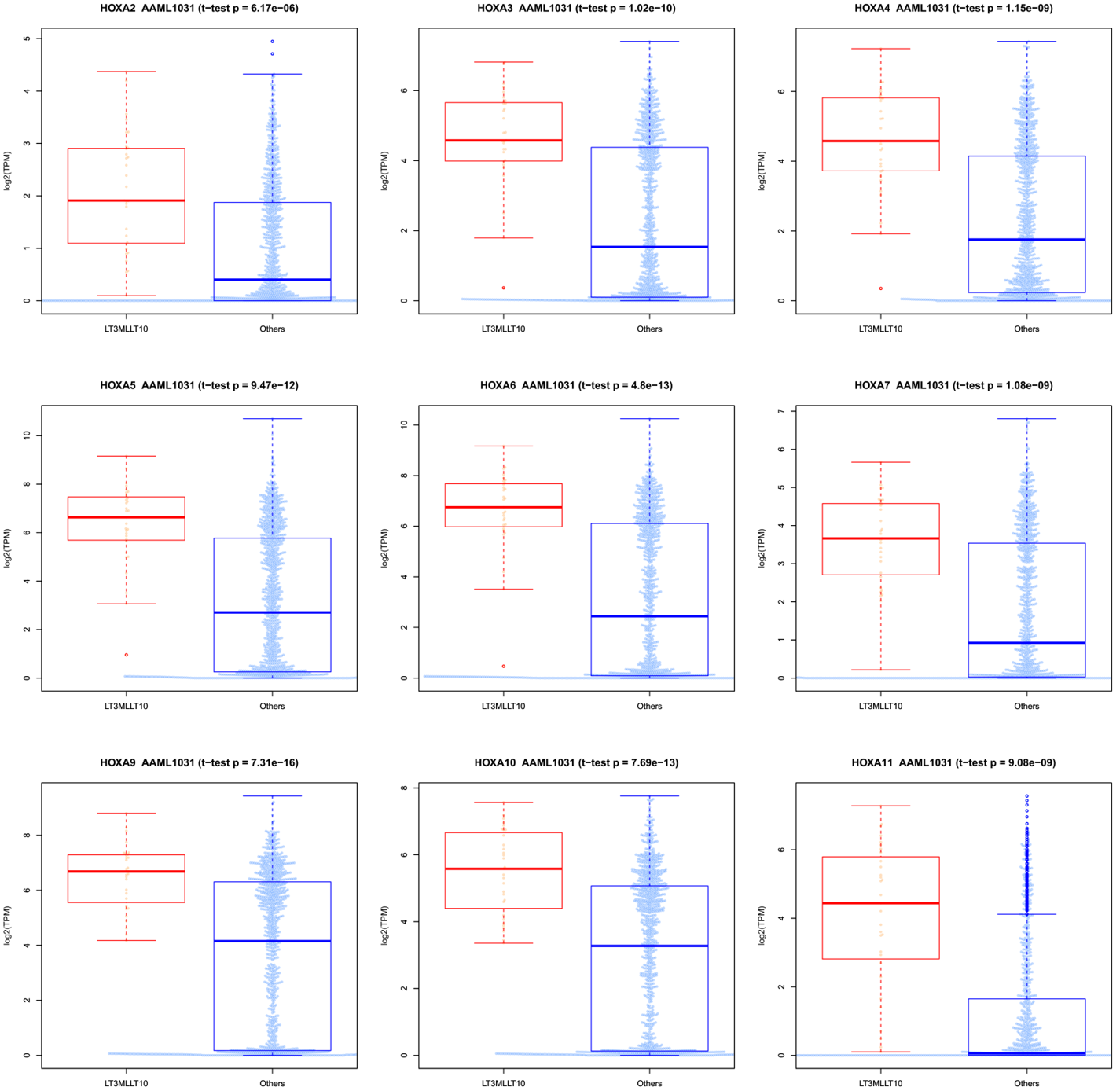

Supplement: S1 Fig — (TIF) [file pone.0259197.s001.tif]

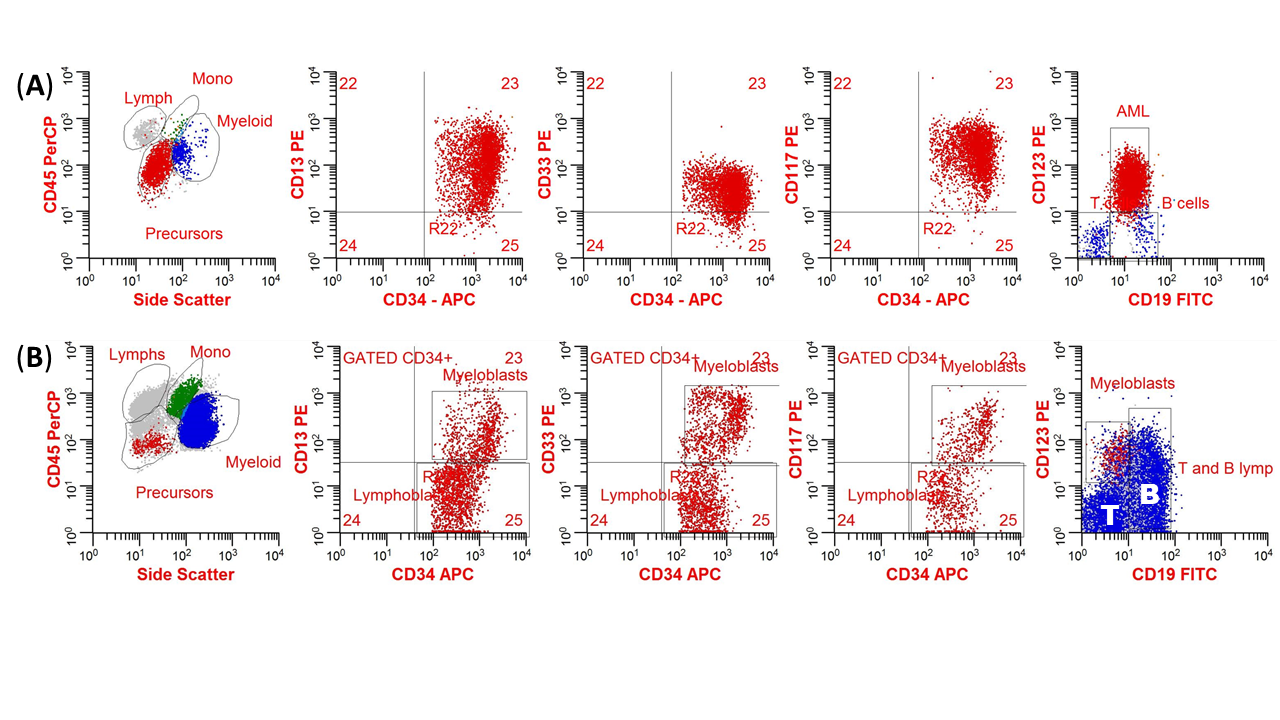

Supplement: S2 Fig — (A) Left to right: CD45/Side Scatter plot shows cell composition of the specimen with abnormal blasts in red, normal neutrophils in blue and normal lymphocytes in grey. Blast cells (in red) were gated to show expression of the stem/progenitor marker CD34, and myeloid associated markers CD13, CD33 and CD117. The last panel shows CD19 and CD123 expression of the leukemic blast cells in red, relative to normal lymphocytes (B lymphocytes CD19 positive) and T lymphocytes (CD19 negative) in blue. (B) Left to right: CD45/Side Scatter plot showing composition of a normal BM specimen with CD34+ myeloid and lymphoid precursors in red, neutrophils in blue, monocytes in green and lymphoid precursors and mature lymphocytes in grey. CD34+ precursors (red) were gated and the expression of myeloid associated antigens CD13, CD33 and CD117 displayed vs CD34. Note there is no expression of either of these antigens on CD34+ lymphoblasts. The last plot shows CD19 and CD123 expression of CD34+ myeloblasts in red relative to normal T and B lymphocytes. (TIF) [file pone.0259197.s002.tif]
